# Multiscale metabolic mapping of lung tissue via coregistered mass spectrometry and nonlinear optical imaging

**DOI:** 10.1126/sciadv.aec3544

**Published:** 2026-07-08

**Authors:** Brittney L. Gorman, Zhi Li, Gail Deutsch, Heidie L. Huyck, Niana Beishembieva, Harsh Bhotika, Heather Olson, Jorge Villazon, Ping Yu, Gloria S. Pryhuber, Geremy Clair, Lingyan Shi, Christopher R. Anderton

**Affiliations:** ^1^ Environmental and Biological Sciences Directorate, Pacific Northwest National Laboratory, Richland, WA 99354, USA.; ^2^ Shu Chien-Gene Lay Department of Bioengineering, University of California, San Diego, La Jolla, CA 92093, USA.; ^3^ Seattle Children’s Hospital, Seattle, WA 98105, USA.; ^4^ Department of Pediatrics, University of Rochester Medical Center, Rochester, NY 14642, USA.

## Abstract

The lung is a highly heterogeneous organ that is composed of numerous microanatomical units, each essential for maintaining intricate functions that work in concert. Disruptions in the molecular and cellular mechanisms can cause tissue fibrosis, inflammation, and severe breathing difficulties, which are common characteristics of the disease of prematurity, bronchopulmonary dysplasia (BPD). BPD’s molecular changes are not well understood, and this has hindered effective diagnosis and treatment. Here, we present a multimodal imaging workflow for detailed molecular and metabolic characterization of lung tissue at multiple spatial scales. We also developed a hierarchical multimodal registration network for precise coregistration of the data from each modality. Our results show that this approach can reveal previously unknown metabolic changes in distinct functional tissue units affected by disease, including altered lipid distributions, reduced optical redox states, and collagen remodeling. This multimodal approach provided detailed maps of molecular shifts occurring in distinct microanatomical features that, when adopted to interrogate this and other tissue types, has the potential to enable the discovery of new therapeutics.

## Teaser

A multimodal imaging approach using advanced spectroscopy and mass spectrometry to identify molecular mechanisms in lung tissue.

## INTRODUCTION

Bronchopulmonary dysplasia (BPD) is a chronic lung disease that primarily affects premature infants, characterized by incomplete lung development and alveolar simplification (*1*). BPD represents the predominant respiratory morbidity in preterm infants, annually affecting ~10,000 to 15,000 neonates in the United States (*2*, *3*). Although BPD is the leading cause of morbidity among premature infants, limited treatment options exist (*4*). The lung’s heterogeneity and the complex pathophysiology of BPD necessitate a comprehensive understanding of the metabolism within tissues to identify potential therapeutic targets and improve patient outcomes (*5*).

Traditionally, researchers have relied on biochemical assays, histological studies, and bulk tissue analyses to investigate metabolic changes, such as altered metabolism of sugars, lipids, and amino acids in the BPD-affected lung (*5*–*10*). Recently, single-cell transcriptomics performed on BPD-dissociated cells revealed the existence of an aberrant capillary cell state, uniquely found in some patients with BPD (*11*). Although these approaches have provided valuable insights, they often lack the combination of spatial information and molecular specificity necessary to fully elucidate the intricate metabolic changes occurring across the diverse anatomical regions of the lung. As an example, spatial proteomics, using highly multiplexed immunofluorescence (MxIF), revealed the loss of alveolar type 1 (AT1) cells, endothelial/capillary cells, and lymphatics, as well as an increase in alveolar type 2 (AT2) cells, smooth muscle, and fibroblasts (*12*). Although important to define changes in cell populations, MxIF, due to its targeted nature, can only profile predefined protein markers and, therefore, provides indirect and limited information on the metabolic changes occurring in the tissue. To address these limitations, several metabolic imaging technologies have emerged as powerful tools for untargeted spatial analysis of metabolites (including lipids) in biological tissues, such as the lung (*13*, *14*). Moreover, the application of different metabolic imaging approaches in a multimodal manner can overcome the limitations of using a singular imaging assay, where they can be used to provide complementary information (*15*).

Matrix-assisted laser desorption/ionization mass spectrometry imaging (MALDI-MSI) has been widely applied to map metabolites and identify biomarkers within tissues (*16*–*18*). MALDI-MSI is particularly advantageous due to its ability to detect many analytes simultaneously in an untargeted manner while also providing precise molecular specificity (*17*, *19*). It enables the identification of individual molecular species and offers detailed insights into molecules’ localization within distinct anatomical regions (*20*, *21*). Although MALDI-MSI can regularly achieve spatial resolutions below 10 μm, subcellular analyses remain challenging. Moreover, MALDI-MSI cannot provide histological information about tissues. Consequently, MALDI-MSI analyses of samples are often supplemented, in a multimodal manner, with light-based imaging methods (*22*–*24*).

Recently, an ultrafast focused light-based imaging and photonics (U-FLIP) platform, which combines two-photon fluorescence (TPF), second harmonic generation (SHG), and stimulated Raman scattering (SRS) into a single microscope, has emerged as a promising method for advanced tissue imaging (*25*–*28*). This label-free optical imaging platform enables visualization of tissue morphology and can capture metabolic activity at subcellular resolutions. By detecting molecules, such as reduced form of NAD^+^ (NADH), flavin adenine dinucleotide (FAD), and collagen, and molecular classes, such as lipids and proteins, this approach can provide critical insights into metabolic and tissue morphological alterations and offers complementary information to the specific molecular annotations provided by MALDI-MSI.

Here, we introduce an integrated workflow combining U-FLIP, MALDI-MSI, and histology. We applied these methods for the complementary spatial analysis of distinct anatomical regions in human lung tissue affected by BPD. As part of this effort, we developed a hierarchical multimodal registration network (HiMReg) to align multimodal data and permit the integrated analysis of MALDI-MSI with U-FLIP. This integration presents a unique opportunity to bridge the gap between molecular specificity and high-resolution morphology. By leveraging the strengths of both technologies, we demonstrate this approach’s ability to characterize the lipidomic composition of alveoli, bronchioles, and vessels identified through histological assessment and correlate these findings with changes in lipid saturation and optical redox at high spatial resolutions. Ultimately, this approach could be further used to illuminate unknown metabolic changes in distinct cells and cell clusters affected by BPD as part of an effort to develop improved therapeutics.

## RESULTS

### Cross-domain registered multimodal imaging workflow

Understanding complex and heterogeneous diseases such as BPD requires a comprehensive spatial characterization of tissue heterogeneity across various molecular and structural dimensions. However, integrating varying resolutions, cross-domain multimodal imaging while preserving spatial relationships has remained a major challenge in biological research. Here, we demonstrate a multimodal imaging workflow and a image registration approach, enabling multitechnique correlations between molecular distributions, metabolic states, and tissue architecture within the same pulmonary tissue sections.

**Fig. 1.**
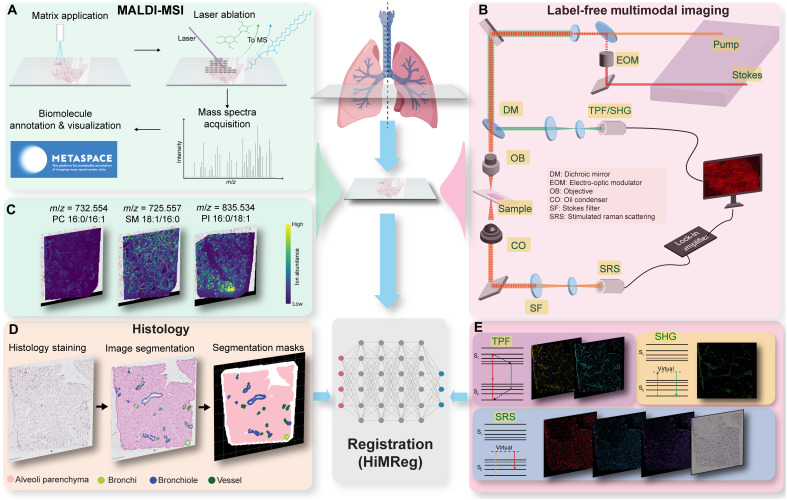
Coregistered multimodal imaging workflow for comprehensive human lung tissue analysis. (**A**) MALDI-MSI approach, including matrix application via an automated sprayer, laser ablation for mass analysis of generated ions, and data processing using METASPACE for biomolecule annotation and visualization. (**B**) Schematic of the U-FLIP imaging platform combining TPF, second SHG, and SRS in one microscope platform. (**C**) Representative MALDI-MS images showing the spatial distribution of specific lipids in the human lung: PC (16:1/16:0), SM (16:1/16:0), and glucosylceramide PI (16:0/18:1). (**D**) Workflow for histological analysis. H&E staining is performed on the same tissue section after label-free multimodal imaging. A pathologist carefully segmented the following FTUs: alveoli parenchyma (pink), bronchi (green), bronchioles (blue), and vessels (dark green). A registration network (HiMReg) integrates cross-modality images for comprehensive spatial analysis. (**E**) Representative images obtained from each modality in (C), where TPF captures NADH and FAD autofluorescence, SHG visualizes collagen fibers, and SRS provides chemical bonding information specific to proteins and lipids.

Our method combines MALDI-MSI for spatial lipidomics and ultrafast focused light-based imaging for metabolic imaging and histological assessment (Fig. 1). This multiscale analysis workflow helped to decipher the complex pathophysiology of the lung, where cellular metabolism, lipid composition, and tissue architecture are intricately correlated. A technical barrier in developing this workflow was cross-modality substrate compatibility. Although conventional MALDI-MSI applications typically use electrically conductive indium tin oxide (ITO)–coated slides for optimal sensitivity, these coated substrates generated interfering nonlinear optical effects that compromised the transmission-based laser setup for SRS measurements (fig. S1).

We found that analyzing serial sections, where consecutive tissue sections were placed on ITO-coated and normal glass slides, was sufficient for individual quantitative analyses. However, this approach was inadequate for precise spatial correlation across modalities as it is extremely difficult to obtain two serial sections without substantial morphological and cell type changes, especially when the sections are generated from pulmonary tissues with a large proportion of open, air-filled space. Therefore, it is ideal to perform multimodal imaging on the same section to enable full integrability of datasets. We optimized MALDI-MSI parameters for nonconductive glass slides by increasing the number of laser shots slightly and adding double-sided conductive copper tape at the edges of the slide to prevent signal suppression caused by charge accumulation. This maintained a high signal-to-noise ratio while minimizing tissue damage. The use of glass slides allowed true same-section multimodal imaging, where MALDI-MSI was performed on the tissue section first, followed by U-FLIP and histology staining.

We applied MALDI-MSI (Fig. 1A) and annotated the resulting molecular profiles using METASPACE against the SwissLipids and our internal lung liquid chromatography–tandem mass spectrometry (LC-MS/MS) databases created using 200-μm tissue sections from the same tissue blocks. This revealed distinct lipid distributions within the lung tissue (Fig. 1C). Post–MALDI-MSI, the matrix was removed, and the tissue sections were chemically fixed using paraformaldehyde. The same tissue section was then imaged by U-FLIP, which combines TPF for metabolic imaging, SHG for collagen visualization, and SRS for chemical composition analysis (Fig. 1, B and E). Last, hematoxylin and eosin (H&E) staining enabled detailed histological assessment and manual annotation of broad functional areas, so-called functional tissue units (FTUs), alveolar parenchyma, bronchioles, bronchi, and large vessels (i.e., vasculature excluding the capillaries located in the alveolar parenchyma), for downstream quantitative analyses (Fig. 1D).

However, achieving precise spatial registration between these diverse imaging modalities presented a substantial challenge, particularly due to image distortion and disparate spatial resolutions. To address this, we developed HiMReg, which is an innovative registration framework to coregister all our imaging modalities’ data (fig. S2A). This approach uses H&E-stained images as a common reference point (i.e., the anchor image that we registered with all other data). Although MALDI-MSI data are regularly aligned with H&E (*24*, *29*), the registration between high-resolution U-FLIP and H&E images posed more substantial technical hurdles. HiMReg first used hierarchical affine registration to establish global positioning, followed by hierarchical diffeomorphic registration to account for local, nonrigid tissue deformations. The network’s multiscale processing approach, implemented through PyTorch-based GPU acceleration (*30*), minimized local minima effects while efficiently handling large-scale image processing. Alignment evaluation demonstrated HiMReg notably outperforms traditional registration methods like Elastix (*31*) (figs. S2, B and C, and S3), achieving superior alignment accuracy as measured by both mutual information (MI) (*32*) and dice coefficient metrics (*33*) (fig. S2, D and E). These gains were consistent across the diverse tissue contexts (fig. S3), supporting that HiMReg is more accurate and more robust than a conventional registration baseline and Elastix.

### Spatial lipidomics of lung tissue

**Fig. 2.**
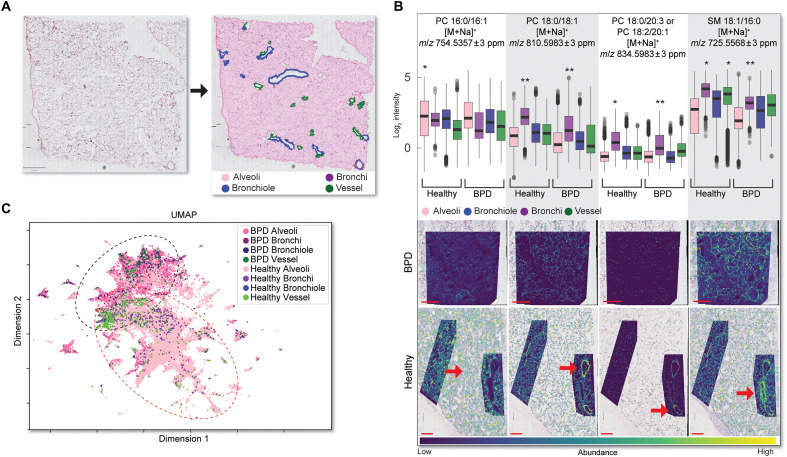
Multivariate analysis of spatial lipidomics of healthy and BPD lung tissue. (**A**) H&E-stained tissue section and QuPath segmented alveoli, bronchi, bronchioles, and vessels. (**B**) Box plots and corresponding ion images showing the distribution of lipids within distinct FTUs of healthy and BPD lung tissue. Red arrows indicate the position of the corresponding FTU where each ion is statistically elevated (*Log_2_ ≥ 0.5; **Log_2_ ≥ 1). ppm, parts per million. Scale bars, 1 mm. (**C**) UMAP analysis of the lipid annotations shows separation between healthy and BPD lung FTUs. The most distinct separation in this multivariant analysis was between the alveoli, encircled by dashed lines.

MALDI-MSI was used on each lung tissue section to obtain lipid composition in an untargeted manner. Multiple tissue blocks (*n* = 4) were selected from the left upper lobe of a healthy lung and spatially matched with blocks from a BPD-affected lung, with an attempt to match for postnatal age corrected for the premature birth (CA) of the BPD lung (~3 months versus 4 months CA; table S1). Tissue sections were first assessed using autofluorescence microscopy to locate regions of interest (ROIs) to the four FTUs described above (i.e., parenchyma, bronchioles, bronchi, and large vessels) (fig. S4). For each tissue, ROIs were selected containing at least one bronchi or bronchiole, vessel, and alveoli-containing region. MALDI-MSI data were acquired within each ROI at a 35-μm lateral resolution to permit the identification of distinct lipid distributions within each airway, vessel, and alveolar region (Fig. 2B).

Leveraging coregistered histology with our MALDI-MSI data, we created histology-based assessments of the lipid populations in each FTU of interest. By graphing each analyzed pixel on a uniform manifold approximation and projection (UMAP), we observed clustering of each FTU (Fig. 2C). However, disease-related differences were less evident. The alveoli-containing pixels (pink) in the healthy and BPD samples separated the most, indicating the presence of more substantial disease-dependent lipidomic differences in the alveoli compared to the other FTUs. To elucidate the lipids influencing these differences, we performed Student’s *t* tests, comparing the ion abundances within each segmented FTU to all other regions in the lung (tables S2 to S4). We first identified representative lipid abundances within each FTU of the healthy tissue and noted consistent abundances of these same lipids in the diseased tissues. In Fig. 2B, representative lipids are shown, where phosphatidylcholine (PC) 16:0/16:0 was most abundant in the alveoli, PC 18:0/18:1 was more abundant in the bronchi and bronchioles, and sphingomyelin (SM) 18:1/16:0 was mostly present in large vessels.

**Fig. 3.**
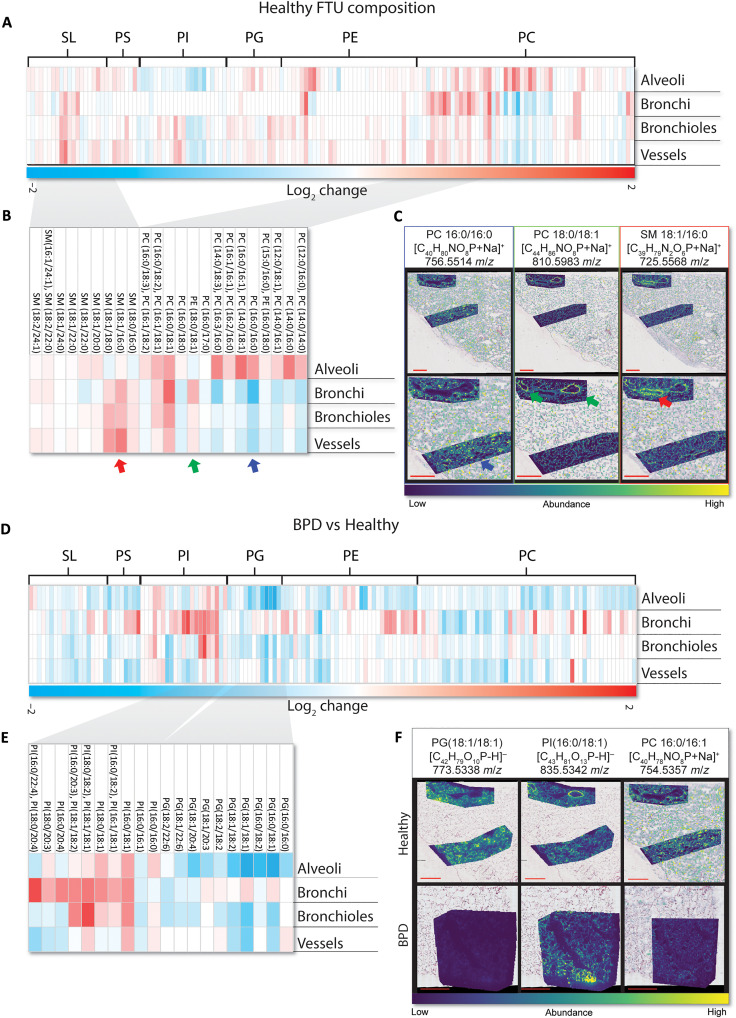
MALDI-MSI lipid profiles of healthy and BPD anatomical regions. (**A**) Heatmap showing the relative abundance of select lipids within each segmented healthy FTU organized by lipid subtype and increasing fatty acid chain length from top to bottom. (**B**) Specific lipid annotation information for a selection of PC and SM lipids. (**C**) MALDI-MSI images showing the relative abundances of PC 16:0/16:0, PC 18:0/18:1, and SM18:1/16:0. PC 16:0/16:0 are the most abundant in the alveoli (blue arrow), PC 18:0/18:1 increased in the bronchi (green arrow), and SM 18:1/16:0 displayed the highest association with the vasculature (red arrow). (**D**) Heatmap showing the relative change in abundance between healthy and BPD tissues within each segmented FTU organized by lipid subtype and increasing fatty acid chain length from top to bottom. (**E**) Specific lipid annotation information for a selection of PG and PI lipids. (**F**) MALDI-MSI images show the relative abundances of PG 18:1/18:1, PI 16:0/18:1, and PC 16:0/16:1. Scale bars, 2 mm.

Further exploration of the lipidomic signatures was done at increasing levels of chemical specificity for each FTU and comparing the healthy FTUs to the BPD ones. Figure 3A shows the relative abundances of the lipids organized by their subtype in each FTU. Here, we observed increased abundances of various PCs in the alveoli and bronchi. Meanwhile, the annotated blood vessels displayed lower abundances of several PCs and increased abundances of sphingolipids (SLs). In particular, as shown in Fig. 3B, we observed that PC 16:1/16:0, PC 14:0/16:0, and PC 16:0/16:0 were most abundant in the alveoli (blue arrow). The bronchi and bronchioles displayed higher abundances of PC 18:0/18:1 and PC 16:0/18:1 (green arrow). The SM 18:1/16:0 was the most intense ion identified within the vessels (red arrow). Last, representative ion images for each region are shown in Fig. 3C.

When compared to the healthy tissue, the BPD lung tissue displayed an overall decrease in PCs, particularly in the alveoli (Fig. 3, D and E). We also observed a decrease in phosphatidylglycerols (PGs). The bronchi and bronchioles of the BPD lung tissue displayed the highest abundances of PCs with longer fatty acid chains. In the airways of the BPD tissue, we observed an increase in phosphatidylinositol (PI) and phosphatidylethanolamine (PE) lipids. These exploratory lipidomic findings insinuate complex metabolic changes in both alveolar and airways regions, with more pronounced changes in the alveolar parenchyma. This suggests the need for broader metabolic mapping of additional BPD and healthy, age-matched lung tissues.

### Metabolic and morphological imaging of lung tissue

To elucidate the metabolic processes not evident through spatial lipidomics alone, we sought to use U-FLIP, which enabled the visualization and quantification of key metabolic indicators and structural components (*34*, *35*) across FTUs in healthy and BPD-affected lung tissues at high spatial resolutions. The nondestructive nature of MALDI-MSI has been routinely capitalized upon to permit histological imaging, as well as immunofluorescence, post–MALDI analysis (*36*, *37*). To verify compatibility of U-FLIP with MALDI-MSI, we first verified that the steps required for spatial lipidomics imaging, such as matrix deposition, laser ablation, and matrix removal, did not affect the quality of the U-FLIP imaging data. First, using a single tissue section, we directly compared U-FLIP spectra from MALDI-analyzed regions with adjacent, non-MALDI–analyzed regions. This test verified that no major imaging artifacts were induced by MALDI laser ablation (fig. S5, B and C). Next, we used two consecutive sections and imaged one with both MALDI and U-FLIP and the other with U-FLIP only. Using this approach, we compared signal attenuation across the FTUs and found no significant spectra differences (fig. S5, D and E), confirming that matrix deposition and removal did not affect U-FLIP results nor biological interpretability.

**Fig. 4.**
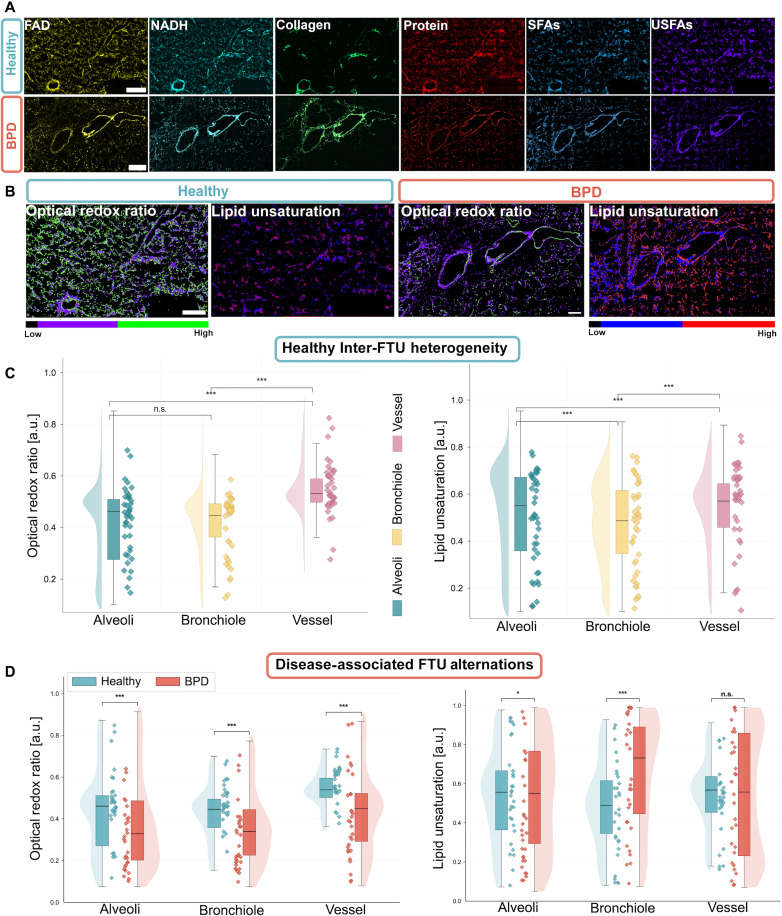
Label-free U-FLIP analyses reveals metabolic and structural differences between healthy and BPD lung tissues. (**A**) Representative label-free U-FLIP images of FAD, NADH, collagen (SHG), protein, SFAs, and USFAs in healthy (top) and BPD (bottom) lung tissue. (**B**) Optical redox ratio and lipid unsaturation maps in healthy (left) and BPD (right) lung tissue. (**C**) Quantification of the optical redox ratio (left) and lipid unsaturation (right) across segmented FTUs (alveoli, bronchiole, and vessel) in healthy tissue, revealing inter-FTU metabolic heterogeneity. a.u., arbitrary units. (**D**) Disease-associated FTU alterations: comparison of the optical redox ratio (left) and lipid unsaturation (right) between healthy and BPD tissue across FTUs. *P < 0.05; ***P < 0.001, n.s., not significant.

Our U-FLIP focused on several critical endogenous molecules involved in metabolism: FAD, NADH, saturated fatty acids (SFAs), and unsaturated fatty acids (USFAs), as well as the protein content and morphological profiles of collagen fibers (Fig. 4A). The normalized optical redox ratio, defined herein as FAD/(NADH + FAD), was obtained from TPF images of said molecules (*38*), and lipid unsaturation ratio, defined as USFAs/(SFAs + USFAs), was derived from SRS ratiometric images of SFAs and USFAs at 2880 and 3011 cm^−1^, respectively (*39*). The optical redox ratio can be used as an indicator of cellular metabolic state and may reflect primarily mitochondrial metabolism as the tricarboxylic acid (TCA) cycle is the major contributor to cellular NAD^+^/NADH and FAD/FADH_2_ pools (*40*, *41*). Therefore, changes in this ratio potentially offer insights into alterations in mitochondrial function and cellular energy metabolism in diseased states (*41*). We investigated each spatial metabolic indicator in the segmented FTUs of healthy human lung tissue (Fig. 4, B and C) and detected significant variations across FTUs through quantitative analysis of the optical redox ratio (Fig. 4C). In the control tissue, the annotated vessels exhibited a higher optical redox ratio than the alveoli and bronchioles, suggesting higher energy demand and oxidation in these regions. Another explanation is that lower FAD(H)/NAD(H) pools persist in vascular structures. Concurrently, alveolar and vascular regions displayed a relatively higher degree of unsaturation compared to bronchioles (Fig. 4C).

Analysis of healthy versus BPD tissues revealed significant metabolic differences related to disease (Fig. 4, A, B, and D). The optical redox ratio analysis (Fig. 4, B and D) displayed a significant decrease in the BPD tissue across all FTUs, suggesting either an alteration of the redox state or an overall reduction of the energy metabolism of the cells. Lipid unsaturation analysis comparison between healthy and BPD (Fig. 4, B and D) was more intricate. Although lipid unsaturation in the alveoli was decreased compared to healthy tissue, it was increased in bronchioles. Nevertheless, metabolic changes in the lung remained difficult to fully decipher at the resolution of SRS.

**Fig. 5.**
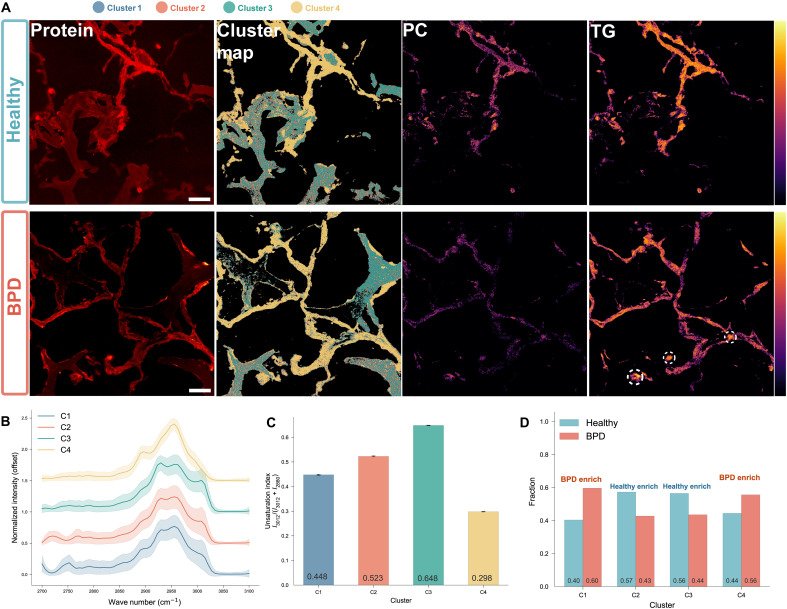
Spatial chemical profiling reveals distinct molecular compositions in healthy and BPD alveoli through hSRS. (**A**) SRS protein images (2930 cm^−1^), *k*-means cluster maps (*k* = 4), and PRM-SRS PC and TG maps for healthy (top) and BPD (bottom) alveoli. (**B**) Normalized Raman spectra (2700 to 3100 cm^−1^) for each cluster showing distinct chemical compositions. (**C**) Lipid unsaturation index for each cluster. (**D**) Fractional cluster composition between healthy and BPD, with enrichment annotations. Scale bars, 50 μm.

To characterize these metabolic alterations with higher spatial granularity, we used hyperspectral SRS (hSRS) with computational approaches to analyze the molecular composition of alveoli at subcellular resolutions. After variance-based feature selection of the top 30% most variable spectral channels and principal component denoising, *k*-means clustering (*k* = 4, optimized via silhouette analysis and spectral interpretability in fig. S6) identified four chemically distinct spectral populations. Although these spectral regions encompassed both healthy and BPD tissues, they were differentially enriched in healthy versus BPD alveoli (Fig. 5, A and D). Cluster-specific Raman spectra revealed that healthy-enriched clusters (C2 and C3) exhibited higher lipid unsaturation content, whereas BPD-enriched clusters (C1 and C4) showed reduced USFA signatures (Fig. 5, B and C). Furthermore, using our penalized reference matching (PRM)–SRS approach (*42*), which is a label-free lipid subtype detection method, we also observed decreased PC levels and increased triglyceride (TG) accumulation in BPD alveoli (Fig. 5A).

**Fig. 6.**
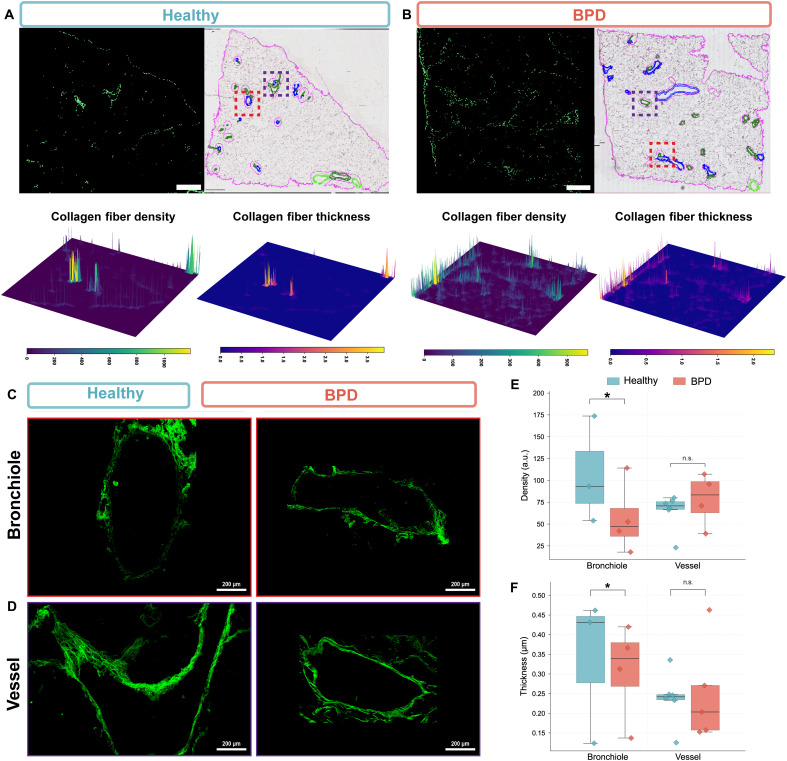
Collagen fiber analysis in healthy and BPD-affected lung tissues. (**A** and **B**) Representative SHG images and corresponding H&E stains of healthy (A) and BPD (B) lung tissues. Scale bars, 1 mm. Collagen fiber density and thickness heatmaps are shown below. (**C**) High-magnification SHG images of bronchioles in healthy (left) and BPD (right) tissues. Scale bars, 200 μm. (**D**) High-magnification SHG images of vessels in healthy (left) and BPD (right) tissues. Scale bars, 200 μm. (**E**) Quantitative comparison of the collagen fiber density in bronchioles and vessels between healthy and BPD tissues. (**F**) Quantitative comparison of the collagen fiber thickness in bronchioles and vessels between healthy and BPD tissues. Data are presented as means ± SD. *P < 0.05, n.s., not significant.

Next, we investigated the structural consequences of BPD on the extracellular matrix. Collagen fibers play a crucial role in maintaining the structural integrity and mechanical properties of lung tissue. They are particularly sensitive to changes in cellular metabolism and oxidative stress (*43*). Using SHG microscopy to visualize collagen architecture, we observed that the BPD lung tissue exhibited changes in collagen ultrastructure (Fig. 6, A and B). Detailed investigation (Fig. 6, C and D) revealed distinct patterns of collagen reorganization in the different FTUs of the BPD lung. In healthy tissue, collagen appeared densely packed with well-organized fibrillar structures and concentrated distribution patterns. In contrast, BPD tissue showed more diffuse collagen arrangements, suggesting altered matrix organization. Quantitative analysis demonstrated a significant reduction in organized bronchiole-associated collagen fiber density as measured by SHG microscopy in BPD compared to healthy tissue (Fig. 6E). This finding likely reflected alterations in collagen organization rather than a decrease in total collagen content, as a previous study showed increased collagen subunit abundance in these regions (*12*). The reduced SHG signal suggested a shift from well-organized collagen fiber structures to more disorganized collagen deposition patterns in BPD tissue. Vascular-associated collagen displayed remarkably different patterns. Despite the pronounced changes in bronchiole regions, both the density and thickness of vessel-associated collagen remained unchanged between healthy and BPD tissues (Fig. 6, E and F). In summary, the comprehensive metabolic and structural information provided by the multiple components of the U-FLIP platform complement the spatially resolved molecular data that MALDI-MSI can offer.

### Coregistered MALDI-MSI and U-FLIP correlation

**Fig. 7.**
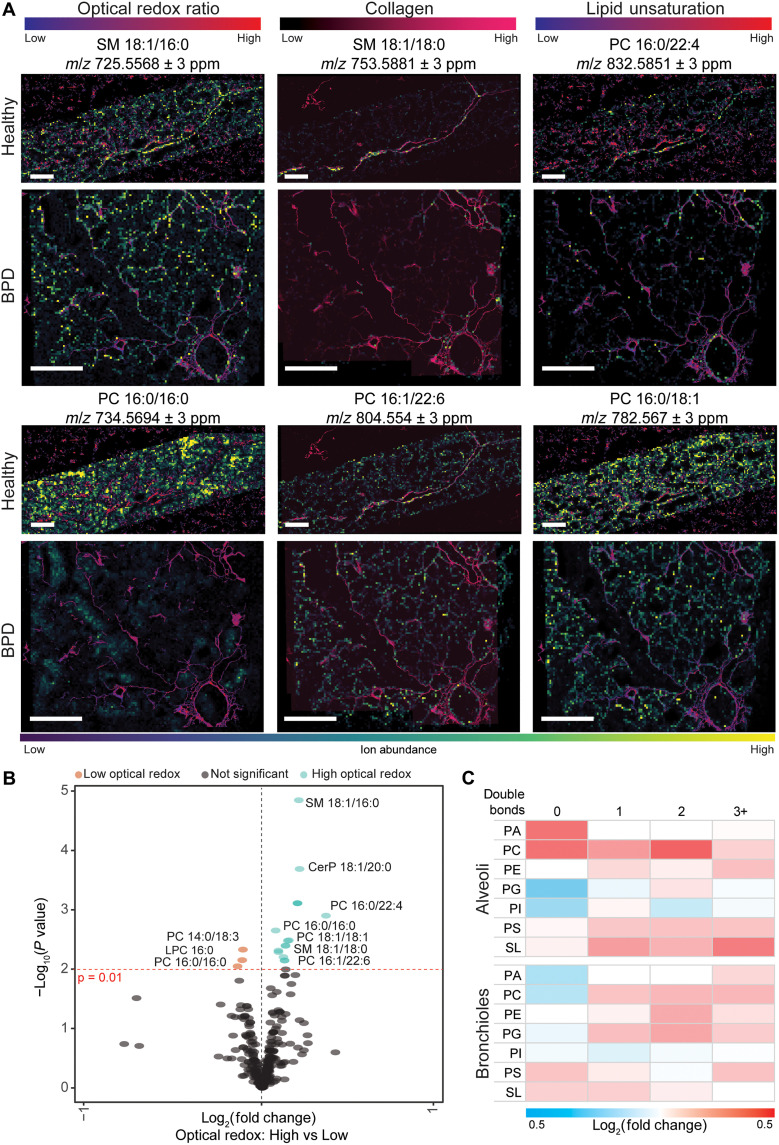
Correlated analysis of coregistered MALDI-MSI and U-FLIP. (**A**) Coregistered multimodal images of the optical redox ratio, collagen, and lipid unsaturation and representative MALDI-MS images of lipid distributions in the same regions. The optical redox ratio is higher in the vasculature of the tissue than the tissue and displays lower redox in the alveoli regions. This corresponded to a similar increase in SM in the vasculature and increased PC 16:0/16:0 in the alveoli regions. The collagen images show the presence of collagen-rich airways and septa in the lung tissue that corresponded with SM 18:1/18:0 and PC 16:0/18:1. The lipid unsaturation signals provide high-resolution information concerning lipid saturation and corresponded with high unsaturation near PC 16:0/22:4 and low unsaturation near PC 16:0/18:1. Scale bars, 500 μm. (**B**) The volcano plot shows the ions that increase in low- and high-optical redox regions (*P* ≤ 0.01). Ten molecules had significantly increased abundance in high-redox regions, and three molecules were increased in low-redox regions. (**C**) Heatmaps compare the abundance of double bonds in the alveoli and bronchioles compared to all other FTUs. The alveoli contain higher abundances of unsaturated PAs and PCs and unsaturated SLs. The bronchioles displayed slight increases in unsaturation in all lipid species.

Our image registration method developed and used here has multiple major benefits. First, it permits alignment of all imaging modalities with histology images and multimodal image segmentation. Second, coregistration enables simultaneous observation of high-resolution U-FLIP images and MALDI-MS images of specific lipids. For example, we observed collagen-rich airways associated with specific lipids (i.e., SM 18:1/18:0) (Fig. 7A). Last, it enhances our targeted analyses of the lipidome by providing ROIs based on specific metabolic readouts, such as lipid unsaturation or optical redox ratio.

In addition to the qualitative function of such images, we used the coregistered image modalities to segment MALDI-MSI data and analyze the ROIs for enriched lipids. Using U-FLIP, we detected an increased optical redox ratio in the annotated vessels compared to the bronchioles (Fig. 7A). These registered images allowed us to use the high-redox and low-redox images to optically guide our evaluation of the lipid abundances in relatively high energy metabolism regions. These analyses revealed that 10 annotated lipids displayed increased abundances in high-optical redox regions, whereas 3 lipids increased in abundance in low-optical redox regions (*P* < 0.01). Of the 10 molecules up-regulated in high-redox regions, 5 were SLs, including SM 18:1/16:0, CerP 18:1/20:0, and SM 18:1/18:0. Multiple PCs, including PC 16:0/16:0 and PC 18:1/18:1 that may be associated with the surfactant, were also identified in the high-redox regions (Fig. 7B).

In addition to U-FLIP–guided lipid analysis, this workflow also permits correlated lipid analyses of the segmented FTUs. Using U-FLIP, we detected a relatively higher degree of lipid unsaturation in the alveoli compared to the bronchioles. However, U-FLIP alone does not provide molecular specificity. As such, we could determine the putative lipid identities by MALDI-MSI. As shown in Fig. 7C, when correlated with the MALDI analysis, we identified two classes of lipids that increased in unsaturation in the alveoli, PCs and SLs, which may contribute to alveolar membrane elasticity (*44*), whereas the bronchioles displayed a modest increase in the unsaturation of phospholipids. Notably, the MALDI-MSI methods used here were not optimized for all lipid subtypes, and additional lipid species (e.g., cardiolipins, TGs, or diglycerides) may contribute to the increase in unsaturation observed in the U-FLIP imaging data.

## DISCUSSION

This work establishes a multimodal imaging approach that integrates MALDI-MSI and high-resolution U-FLIP on the same tissue sections to generate multidomain maps that enable evaluation of the complex molecular physiology of lung FTUs in healthy and diseased tissues. The central advantage of this approach is that it couples MALDI-MSI’s molecular specificity with the U-FLIP platform’s higher spatial resolution, broad metabolic readouts, and structural insights without data quality compromise for both methodologies. This facilitates same-section, cross-validation and interpretation of metabolic differences, which is not achievable by either modality alone. Key to the success of this approach is coregistration using HiMReg, which enables FTU-resolved comparisons across the modalities.

Using this workflow, we performed molecular, metabolic, and structural profiling of bronchi, vessels, and alveolar parenchyma regions of human lung tissue. For example, in the annotated vessels, MALDI-MSI and U-FLIP jointly highlighted vessel-specific metabolic signatures. MALDI-MSI indicated high abundances of long-chain PCs and SLs (Fig. 3A), whereas U-FLIP data suggest elevated optical redox ratios (Fig. 4C). Because of our accurate coregistration, we could specifically query MALDI-detected lipids within U-FLIP–defined high-redox regions. Within these high-optical redox regions, we also observed high abundances of SM 18:1/16:0, CerP 18:1/20:0, and SM 18:1/18:0 (Fig. 7B). These SLs are implicated in many cellular processes, including molecular transport, immune response, and signaling (*45*, *46*), and the increased presence of the FAD-to-NADH ratio in the vasculature may reflect the high energy needs of cells in the vessel walls (*47*, *48*). This U-FLIP–driven analysis illustrates a generalizable analysis pattern enabled by this pipeline, where biologically relevant, microenvironments can be molecularly characterized by MALDI-MSI.

Comparison of healthy and BPD tissues further demonstrates how the pipeline can identify specific molecular contributions to dysregulation. MALDI-MSI captured FTU-level shifts in lipid classes and individual lipid species, whereas U-FLIP provided assessment of collagen organization and high-resolution molecular characterization. Within the BPD airways, we observed changes in lipid composition and a decrease in the collagen fiber organization. Specifically, we observed a relatively higher proportion of longer-chain PCs (Fig. 3A) and more FA saturation (Fig. 7C). These longer-chain phospholipids provide increased membrane rigidity (*44*, *49*, *50*). In addition, we observed diffuse and unstructured collagen organization in these BPD airways, potentially due to altered airway cells/basement membrane interactions and airway rigidity (*51*, *52*). This may contribute to the previous observation of reduced passive respiratory system compliance in patients with BPD (*53*). However, because of the limited number of samples analyzed, our histological annotations do not contain enough granularity to provide broader depictions of the tissue. An analysis of a larger number of samples, originating from different donors and topological regions of the organ would be required to gain further granularity and determine whether the chain length of PCs and collagen organization correlates with airway size and BPD pathology.

MALDI-MSI analysis also revealed decreased PCs and PGs found in BPD alveolar parenchyma. We noted decreased abundances of PC 16:0/16:0 and PC 16:0/16:1, which are common in the surfactant. The surfactant is produced by AT2 cells to coat most of the alveolar surface, and due to its high packing density, PC 16:0/16:0 provides the surface tension reduction needed at the air-liquid interface of the distal lung during the breathing cycle (*1*, *54*). In BPD, decreased abundance or function of AT2 cells may lead to changes in the metabolic profile of both the alveolar epithelium and the pulmonary surfactant (*1*). Notably, the inability of MALDI-MSI here to distinguish surfactant from adjacent cellular layers underscores a practical limitation of MSI-only interpretation and motivates the added value of U-FLIP for resolving fine structural layers.

Using U-FLIP, we observed an increase in PCs and lipid unsaturation in the innermost layer of the BPD alveoli (Fig. 5). Many factors can influence the observed changes in lipid composition, including a reduction in surfactant production, tissue remodeling resulting in changes in the availability of circulating lipids during BPD, increased reliance on AT2 for de novo lipid synthesis (*1*, *5*, *12*, *55*, *56*), or an increased prevalence of alveolar macrophages with distinct lipid profile (*57*). However, one limitation of this finding is the potential diffusion and removal of soluble biofluids, such as the mucus and surfactant, during the steps of embedding and solvent washes before U-FLIP. It is advantageous that both the healthy and BPD lung studied were processed by a standard protocol to reduce artifact of tissue preparation. A larger cohort of samples will be needed to determine the variability between multiple healthy lungs and various degrees of severity of BPD. The methods shown in this study lay the groundwork for making these measurements and comparisons possible.

We demonstrate the advantages of using a multimodal imaging workflow to investigate metabolic differences between lung FTUs from healthy and diseased tissue. Beyond qualitative covisualization, the pipeline enables segmentation and cross-modality querying, providing a framework to link broad metabolic state changes to specific lipidomic alterations within the same tissue context. Overall, our workflow provides a method for analyzing broad metabolic changes at high spatial resolutions, and it permits us to link these metabolic shifts to specific lipidomic alterations within tissue sections. In the future, with expanded cohorts, this approach should facilitate systematic mapping of FTU-resolved metabolic remodeling in many diseases.

## MATERIALS AND METHODS

### Lung tissue preparation

Lung tissue was procured through the BioRepository for INvestigation of Diseases of the Lung (BRINDL) (*58*). Four agarose-inflated, carboxymethylcellulose (CMC)–embedded fresh-frozen lung tissue blocks were chosen from the left upper lobe of a donor with BPD and an age-matched control (*59*). Serial tissue sections were cut at 20-μm thickness and thaw mounted on two Superfrost Plus Microscope Slides (Fisher Scientific) and two ITO-coated glass slides (Delta Technology). Additional tissue sections (200 μm) were collected in a 1.7-ml Sorenson tube for lipid extraction and LC-MS analysis.

### MALDI-MSI sample preparation and data acquisition

A detailed MALDI-MSI protocol is available at protocols.io (*60*). Tissue sections were preanalyzed by autofluorescence microscopy using a Zeiss Axio Zoom Microscope. Red (545/572 nm), green (488/509 nm), and blue (353/465 nm) autofluorescence channels were collected along with a bright-field image. ROIs were selected so that at least one airway, muscularized vessel, or alveolar region was analyzed from each tissue section. An M5-Sprayer (HTX Technologies) was used for matrix application, where a N_2_ pressure of 10 psi, a track spacing of 3 mm, and a 40-mm distance between the nozzle and sample were maintained for preparation of all samples. For positive ion mode analysis, 2,5-dihydrobenzoic acid (DHB; 40 mg/ml in 70% MeOH:H_2_O) was sprayed at a rate of 50 μl/min and 75°C for 12 passes. For negative ion mode, *N*-(1-naphthyl)ethylenediamine dihydrochloride (NEDC; 7 mg/ml in 70% MeOH:H_2_O) was applied at 120 μl/min and 75°C for eight passes.

MALDI-MSI analysis was performed using Bruker 12-T Fourier transform ion cyclotron resonance mass spectrometry (FTICR-MS) in positive and negative ion modes. Both modes were tuned using sodium trifluoroacetate (1 μg/ml), and maximal transmission and detections were optimized based on signals in the range of 600–900 mass/charge ratio (*m/z*). The positive ion mode method used 120 laser shots at 2000 Hz and had a mass resolving power of ~180,000 at 400 *m/z*. In negative ion mode, 100 laser shots were applied at 2000 Hz and ~240,000 at 400 *m/z* mass resolving power was achieved. When performing MALDI-MSI on glass slides, the slides were mounted using copper tape on a Bruker target plate and each method was adjusted to use 160 laser shots in positive ion mode and 120 in negative ion mode.

### MALDI-MSI data processing

MALDI-FTICR-MSI data were imported into the Bruker SCiLS Software (version 2024b) and converted into the imzML format. The resulting imzML and ibd files were uploaded to METASPACE (*61*) for automated molecular annotation against the SwissLipids, LipidMaps, and internal LC-MS/MS lung databases (link to data provided in Data, code, and materials availability). Lipid identifications from SwissLipids [false discovery rate (FDR) ≤ 20%] and the custom LC-MS/MS database were downloaded and imported back into SCiLS for data integration and visualization. Of the 280 lipids putatively annotated by the SwissLipids database (FDR ≤ 20%) and confirmed using annotations against an internal LC-MS/MS database, 108 species were present in over 70% of the analyzed regions. These 280 molecular annotations were imported into SCiLS; and histology images and U-FLIP images were aligned with the MALDI images within SCiLS for visualization and analysis.

MALDI-MSI, histology segmentations, and U-FLIP image segmentations were exported through SCiLS API (2024b) and analyzed in “RomicsProcessor” (V1.6), an open-source R package for mass spectrometry data analysis. Where MALDI pixels overlapped with multiple FTUs, we included the pixels in all overlapping groups. Ion intensities were median centered, and a log_2_ transformation was applied. Pseudobulk data reduction was applied to compute the mean abundance of each function tissue unit within each tissue section. This resulted in *n* = 4 for each condition and FTU. A two-tailed Student’s *t* test was performed to compare each FTU (i.e., BPD alveoli, healthy alveoli, BPD bronchi, healthy bronchi, etc.) to all other regions for both the pseudobulk data and unreduced data. Log_2_ fold intensity changes between regions and *P* values were computed for each. Processed data can be visualized on the LungMAP consortium website within ROmics Visualizer (link provided in Data, code, and materials availability).

### Bulk lipidomics

An internal lipid database was created using LC-MS/MS data collected from 200-μm serial tissue sections. Briefly, on each section, the metabolite, protein, and lipid extraction (MPLEx) protocol was performed, as described previously (*62*–*65*). Additional details can be found in the Supplementary Materials. Confident lipid identifications were made using the in-house developed identification software LIQUID, where the tandem mass spectra were examined for diagnostic ion fragments along with associated hydrocarbon chain fragment information (*63*).

### Ultrafast focused light-based imaging and photonics

U-FLIP methods are described in protocols.io (*66*). An upright laser-scanning microscope (DIY multiphoton, Olympus) with a 25× water objective [XLPLN, WMP2, 1.05 numerical aperture (NA), Olympus] was applied for near-infrared throughput. A synchronized pulsed pump beam (tunable 720- to 990-nm wavelength, 5- to 6-ps pulse width, and 80-MHz repetition rate) and a Stokes beam (wavelength at 1032 nm, 6-ps pulse width, and 80-MHz repetition rate) were supplied by a picoEmerald system (Applied Physics & Electronics) and coupled into the microscope. The pump and Stokes beams were collected in transmission configuration by a high-NA oil condenser (1.4 NA). A high-optical-density short-pass filter (950 nm, Thorlabs) was used that would completely block the Stokes beam and transmit the pump beam only onto a Si photodiode for detecting the stimulated Raman loss signal. The output current from the photodiode was terminated, filtered, and demodulated in X with a zero-phase shift by a lock-in amplifier (HF2LI, Zurich Instruments) at 20 MHz. The demodulated signal was fed into the FV3000 software module FV-OSR (Olympus) to form the image during laser scanning. All SRS images were obtained with a pixel dwell time 20 μs and a time constant of 15 μs. The laser power incident on the sample is ~40 mW. SHG was used to capture collagen type 1 to 3 images. The 1031-nm Stokes laser described above, with 300 mW and a dwell time of 8 μs/pixel, was used with three-frame averaging. Backscattered SHG signals were filtered using a 465-nm filter. NADH and flavin autofluorescence images were captured using the 800-nm pump beam with 350 mW and a pixel dwell time of 10 μs/pixel with three-frame averaging. Backscattered signals were filtered using a dual filter cube of 460 and 515 nm. All wide-view tile stitching was controlled by the Fluoview software (Olympus). hSRS images for chemical composition analyses were acquired with a 60-frame image stack, and other parameters are the same as single SRS image.

### Histology imaging

The same sections imaged with MALDI-MSI and U-FLIP when mounted on glass slides were lastly stained by a standard H&E staining protocol. Each slide was rinsed with 70% ethanol (EtOH) and H_2_O for 1 min each. Then, the slides were stained for 30 s in Mayer’s hematoxylin solution (1 g/liter; Sigma-Aldrich). Slides were rinsed with H_2_O and dipped in a bluing solution (American Master Tech Scientific) for 20 s, followed by incubation in H_2_O, 70% EtOH, and 95% EtOH for 30 s each. Last, slides were dipped 4× in eosin (1%, Sigma-Aldrich) and dipped twice in 95% EtOH, twice in 100% EtOH, and xylene for dehydration. Each tissue section was coverslipped and imaged using an Aperio ScanScope XT at a 20× magnification. Images were imported into QuPath (v0.3.8) (*67*), where the alveoli, bronchi, bronchioles, and vessels were manually annotated by a pathologist.

### Hierarchical multimodal registration network

HiMReg implements a two-stage registration optimization combining hierarchical affine and diffeomorphic transformations. The network leverages multiscale from coarse-to-fine processing and GPU acceleration through PyTorch to achieve efficient and accurate alignment of multimodal images. This configuration within PyTorch runs in ~25 s per image pair (3000 by 3000 pixels) on a single RTX A6000, and quantitative benchmarking is shown in fig. S3.

Stage 1: Hierarchical affine registration. The affine transformation $T_{A}$ maps source coordinates x=(x,y) to target coordinates $x^{\prime }$ through

$$x^{\prime }=T_{A}\left(x\right)=Ax+b$$ (1)

where A is a 2 by 2 matrix encoding rotation and scaling, and b is a translation vector. The optimization minimizes

$$L_{\text{affine}}=L_{\text{sim}}\left(I_{\text{fixed}},I_{\text{moving}}\cdot T_{A}\right)+\lambda_{\text{reg}}∥A-I{∥}_{F}^{2}$$ (2)

where $L_{\text{sim}}$ is a similarity metric (MI or local normalized cross-correlation), $\lambda_{\text{reg}}$ is a regularization parameter, and $∥\cdot {∥}_{F}$ denotes the Frobenius norm.

Stage 2: Hierarchical diffeomorphic registration. The diffeomorphic stage refines local deformations through a displacement field v(x). The transformation ϕ maps the spatial coordinates directly

ϕ(x)=x+v(x) (3)

where ϕ(x) represents the spatial transformation mapping initial coordinates x to their deformed positions, and v(x) is the displacement field that defines the deformation. To ensure that the transformation is diffeomorphic (smooth and invertible), we regularized the displacement field gradient and the final optimization objective combines similarity and regularization terms

$$L_{\text{diff}}=L_{\text{sim}}\left(I_{\text{fixed}},I_{\text{moving}}\cdot \phi \right)+\lambda_{\text{smooth}}\int_{\Omega }∥\nabla v{∥}^{2}dx$$ (4)

where the first term measures the similarity between the fixed image and the transformed moving image, whereas the second term enforces smoothness of the displacement field through its spatial gradient. The smoothness weight $\lambda_{\text{smooth}}$ controls the trade-off between image alignment accuracy and transformation regularity to avoid over transformation.

Each registration stage uses a hierarchical approach with L resolution levels. At each level l, images are downsampled. The optimization begins at the coarsest level (highest downsampling factor) and progressively refines the transformation parameters $\theta_{l}$ as the resolution increases

$$\theta_{l-1}=D\left(\theta_{l}\right)+\Delta \theta_{l-1}$$ (5)

where D adapts the transformation parameters from a coarser level l to a finer level l−1, and $\Delta \theta_{l-1}$ represents the refinement at the finer scale. This hierarchical strategy first captures large-scale deformations at coarse resolutions (e.g., 8× downsampling) before refining local details at finer scales (e.g., 4× and 2×), ultimately reaching the original resolution.

Because of the multimodal nature of our registration task, we used MI as the primary similarity metric $L_{\text{sim}}$. MI is particularly suitable for multimodal registration as it makes no assumptions about the intensity relationships between different imaging modalities. The MI between two images *X* and *Y* is defined as

$$\text{MI}\left(X,Y\right)=\sum_{x,y}p\left(x,y\right)\text{log}\frac{p\left(x,y\right)}{p\left(x\right)p\left(y\right)}$$ (6)

where p(x,y) is the joint probability distribution of the intensities in images *X* and *Y*, and p(x) and p(y) are their respective marginal distributions. MI measures the statistical dependency between the intensities of the two images, reaching its maximum when the images are optimally aligned.

The registration evaluation was also assessed using the Dice similarity coefficient (DSC), which measures the spatial overlap between the transformed moving image and the fixed image

$$\text{DSC}=\frac{2|X\cap Y|}{|X|+|Y|}$$ (7)

where ∣X∣ and ∣Y∣ represent the cardinalities of the two segmented regions, and ∣X∩Y∣ is the size of their intersection. The DSC ranges from 0 to 1, providing a quantitative measure of registration accuracy.

### U-FLIP analysis

All image analysis was performed using custom Python scripts. For TPF data, NADH and FAD images were first corrected for background using a threshold-based mask. The normalized optical redox ratio was calculated pixel-wise as FAD/(NADH + FAD). Regions with signal intensity below the noise threshold were excluded from the analysis. SHG images were analyzed to quantify collagen fiber organization using a custom pipeline. Images were preprocessed using Gaussian filtering (σ = 1) to reduce noise while preserving fiber structures. Binary masks were generated using Otsu thresholding, followed by morphological operations to remove small artifacts. Fiber density was calculated as the ratio of collagen-positive pixels to total tissue area. Local fiber thickness was measured using distance transform methods on the skeletonized fiber network. U-FLIP images were also evaluated for signal differences post–MALDI imaging. The detailed methods are available in the Supplementary Materials and shown in fig. S5.

hSRS data were processed using a multistep workflow. Raw spectral data underwent baseline correction to remove water background. Spectra were then normalized using maximum intensity normalization and smoothed with a Whittaker filter (λ = 0.2). *K*-means clustering (*k* = 3) was applied to the processed spectra to identify chemically distinct regions. The lipid unsaturation ratio was calculated from intensity-normalized SRS images as *I*_3011_/(*I*_2880_ + *I*_3011_). For spatial analysis of FTUs, ROIs were manually segmented using the QuPath software. Then, 20,000 pixels from each FTUs were selected randomly for pixel-wise quantitative analysis. At high resolutions, each FTU annotation can contain a large number of pixels, which can make statistical analysis computationally expensive. Pixel-wise sampling is a pragmatic Monte Carlo subsampling method applied to balance computational tractability and equal weighting across FTUs for chemically diverse regions (*68*). Pixels were chosen at random and from each FTU.
