# Supplementary material for: Multiscale metabolic mapping of lung tissue via coregistered mass spectrometry and nonlinear optical imaging
Source: Sci Adv. 2026 Jul 8;12(28):eaec3544. doi: 10.1126/sciadv.aec3544 (PMC13344287; doi:10.1126/sciadv.aec3544)
Supplement: Supplementary file 1 — Supplementary Text Figs. S1 to S6 Table S1 Legends for tables S2 to S5 [file sciadv.aec3544_sm.pdf]

Science Advances
AAAS

Supplementary Materials for

Multiscale metabolic mapping of lung tissue via coregistered mass spectrometry and nonlinear optical imaging

Brittney L. Gorman *et al.*

Corresponding author: Lingyan Shi, l2shi@ucsd.edu; Christopher R. Anderton, christopher.anderton@pnnl.gov

Sci. Adv. **12**, eaec3544 (2026)
DOI: 10.1126/sciadv.aec3544

The PDF file includes:

Supplementary Text
Figs. S1 to S6
Table S1
Legends for tables S2 to S5

Other Supplementary Material for this manuscript includes the following:

Tables S2 to S5

Supplementary Text

Supplemental Methods

Evaluation of HiMReg image alignment

We benchmarked the newly developed HiMReg against conventionally applied Elastix and MatchAnything, as well as the unregistered baseline images using multiple complementary metrics that assess co-registration quality at the pixel/structure level across four representative ROIs (**Figure S3**). Specifically, we report intensity/statistical agreement metrics appropriate for multimodal data (mutual information, MI), edge/structure-focused similarity metrics that reduce sensitivity to modality-specific intensity mappings (GradNCC and GradSSIM, computed on image gradients), and segmentation/boundary-based metrics that quantify spatial correspondence of tissue geometry (TissueDice, TissueIoU, and ASSD). Across ROIs, HiMReg shows uniformly better performance than Elastix on every metric (e.g., MI increases from 0.084 with Elastix to 0.280 with HiMReg; GradSSIM increases from 0.273 to 0.461; TissueDice increases from 0.357 to 0.607; ASSD decreases from 29.8 to 18.2). These gains are consistent across different tissue contexts, supporting that HiMReg is not only more accurate but also more robust and generalizable than a conventional registration baseline. These metrics provide modality-agnostic and structure-sensitive quantitative assessment of co-registration accuracy that is applicable across this multimodal dataset and directly supports confidence in downstream pixel-wise multimodal correlations.

Validation of U-FLIP results post-MALDI-MSI analysis

After MALDI-MSI the matrix was removed and from the tissue sections and the tissues were fixed with paraformaldehyde and washed prior to U-FLIP analysis. To verify that the U-FLIP spectroscopic signals are not attenuated by MALDI-MSI, we performed a within-section control experiment. Shown in **Figure S5**, we selected a single lung tissue section in which only a defined quadrant underwent MALDI-MSI acquisition before the entire section was imaged by U-FLIP. This design provides a direct comparison between MALDI-processed and non-MALDI tissue on the same slide, under identical optical conditions, eliminating inter-section variability as a confounding factor. We examined two U-FLIP channels that are central to the biologically relevant observations: the SRS protein channel (791 nm) and the two-photon optical redox ratio (FAD/(NADH + FAD)). We first applied a MALDI boundary transition analysis (**Figure S5B, C**). This analysis provides the most direct spatial test for processing artifacts, as it compares tissue immediately adjacent on either side of the MALDI acquisition boundary, the same illumination zone, and the same tissue section, where any artifact introduced by MALDI processing would manifest as a sharp intensity step. We rasterized the MALDI polygon into a binary mask on the full field-of-view image and computed a signed Euclidean distance transform, assigning each pixel a distance value (negative inside the MALDI region, positive outside, zero at the boundary). The distance computation is omnidirectional, where for each pixel, the distance is measured to the nearest boundary point regardless of direction, so pixels from all sides of the polygon contribute equally and any localized illumination gradient is averaged out. We divided the distance axis into 100-pixel-wide bands spanning ± 2,000 pixels from the boundary (40 bands total) and computed mean intensity, standard deviation, pixel count, and 95% confidence intervals for each band.

To quantify any step change, we performed a Welch's t-test comparing the 200-pixel band immediately inside the MALDI region against the 200-pixel band immediately outside, supplemented by Cohen's d as an effect-size measure. For the SRS protein channel, the boundary-

adjacent difference was 3.6% with Cohen's d = 0.30 (a small effect by conventional criteria), and importantly, the smoothed trend line showed no abrupt step at the boundary—the variation across the boundary was comparable in magnitude to the natural spatial fluctuation along the entire distance axis. For the optical redox ratio, the difference was 0.3% with Cohen's d = −0.04 (negligible effect), and the intensity profile was essentially flat across the MALDI boundary, demonstrating that MALDI processing has no detectable impact on the optical redox signal.

Next, we performed an intensity comparison (**Figure S5D,E**). We annotated matched functional tissue units—bronchioles, vessels, and alveolar parenchyma in both the MALDI-processed and adjacent non-MALDI regions. For each tissue type and imaging channel, we extracted all tissue pixels within each annotated region and computed kernel density estimates (KDE, Scott's bandwidth) to visualize the full intensity distributions. Percent differences were calculated as |mean_MALDI − mean_nonMALDI| / overall_mean × 100%. For tissue-dense structures (bronchiole and vessel), intensity differences were small across both channels: bronchiole protein 4.5%, bronchiole redox ratio 4.5%; vessel protein 1.3%, vessel redox ratio 3.4%. The alveolar SRS protein channel showed a larger difference (13.8%), which is expected given the inherently low signal intensity and high spatial variability of alveolar parenchyma; critically, the alveolar optical redox ratio—the metabolic indicator used in our biological comparisons—differed by only 1.9%. In all cases, the MALDI and non-MALDI distributions were highly overlapping, indicating no systematic shift attributable to MALDI processing.

U-FLIP Subsampling for Statistical analysis

20,000 pixels randomly selected per FTU for statistical analysis of U-FLIP abundances. This strategy was implemented to balance computational expense, provide equal weight to chemically diverse FTUs, and provide stable estimations of each ROI-distribution. We performed this analysis on a pixel-wise basis because each spatial location (pixel) produces a hyperspectral "datacube". Where downstream quantification is inherently pixel-based and molecular composition can be evaluated at a given pixel location.

At our imaging resolution, a single FTU annotation can contain thousands to millions of pixels; analyzing all pixels is expensive and, importantly, would overweight large FTUs relative to smaller FTUs. Therefore, we cap the per-FTU sample size and draw pixels uniformly and at random from within each FTU mask (excluding background/low-SNR pixels as described in the Methods for redox/unsaturation calculations). We empirically tested multiple per-FTU sample sizes and observed that summary statistics and correlation estimates stabilized while runtime increased substantially beyond this range, motivating the 20,000-pixel cap. Similar random-pixel subsampling strategies are common in imaging when full-pixel analyses are computationally expensive; for example, an SRS imaging study explicitly reports randomly selecting 20,000 pixels due to the long computational time required for correlation-function estimation *(68).*

Supplemental Figures

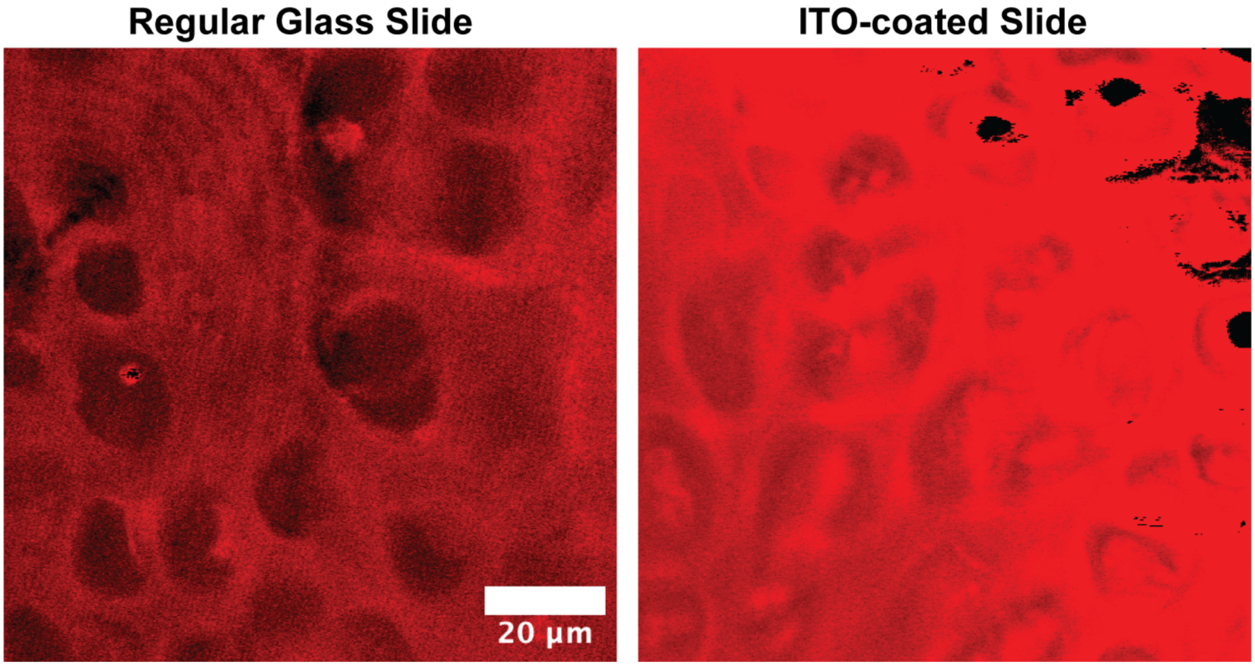

Figure S1. Comparison of SRS protein channel images acquired from human lung sample on regular glass and ITO-coated slide. SRS imaging of the protein channel demonstrates significant differences in image quality between standard glass and ITO-coated slide. Images acquired on ITO-coated slide exhibit consistent oversaturation and substantially reduced signal-to-noise ratio across all Raman shifts.

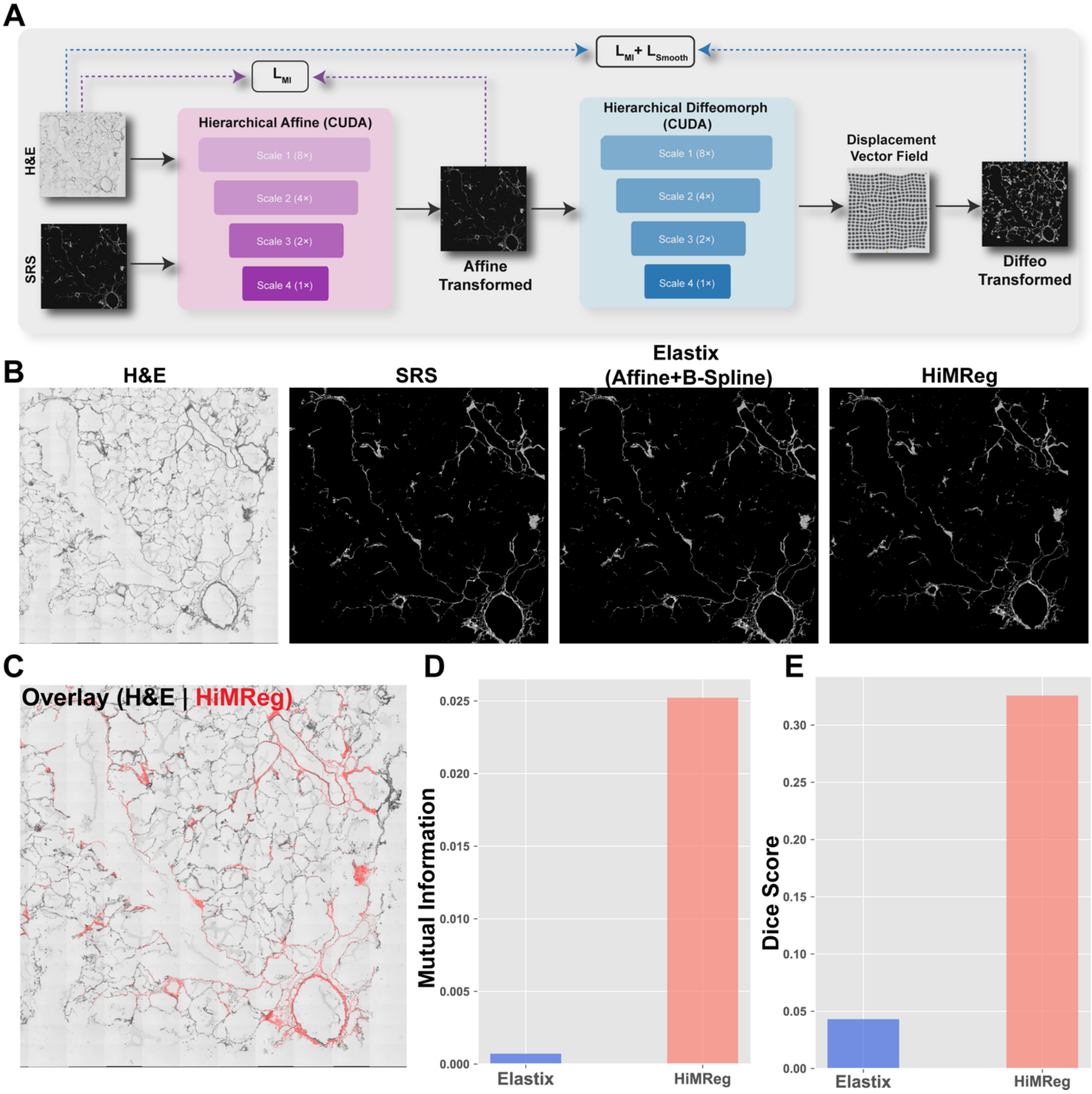

Figure S2. Hierarchical Multimodal Registration network (HiMReg) architecture and performance evaluation. (A) Schematic overview of the HiMReg architecture showing the two-stage registration process: hierarchical affine registration (purple) followed by hierarchical diffeomorphic registration (blue). Each stage employs a customized pyramidal approach with GPU acceleration. **(B)** Visual comparison of registration results between original H&E and SRS images using Elastix (Affine+B-Spline) and HiMReg methods. **(C)** Overlay visualization of H&E (grayscale) and HiMReg-registered SRS (red) images demonstrating precise alignment of tissue structures. **(D,E)** Performance comparison between Elastix and HiMReg using mutual information (D) and dice (E) metrics, showing superior registration accuracy achieved by HiMReg.

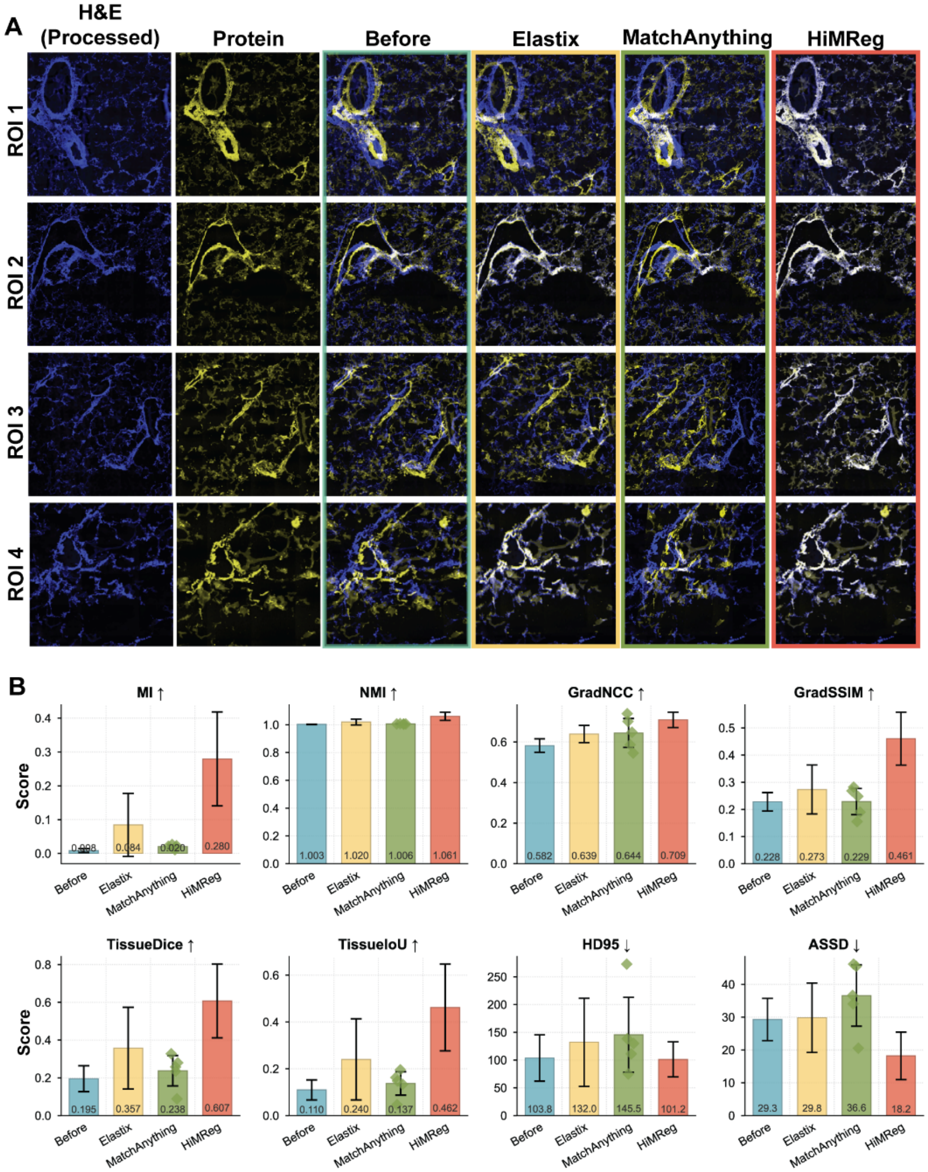

Figure S3. Quantitative validation of HiMReg pixel-level multimodal co-registration accuracy. (A) Representative co-registration examples from four regions of interest (ROIs) aligning the U-FLIP protein channel image (yellow) to the processed H&E image (blue). Overlays are shown before registration ("Before"), after Elastix, after MatchAnything, and after HiMReg; improved co-localization of tissue structures is indicated by increased overlap (appearing bright/white in the overlay). **(B)** Quantitative registration accuracy across ROIs using complementary similarity and geometry metrics: mutual information (MI), gradient normalized cross-correlation (GradNCC), gradient structural similarity (GradSSIM), tissue-mask overlap (TissueDice; TissueIoU), and boundary agreement (average symmetric surface distance, ASSD). Bars show mean ± SD and markers denote individual ROIs.

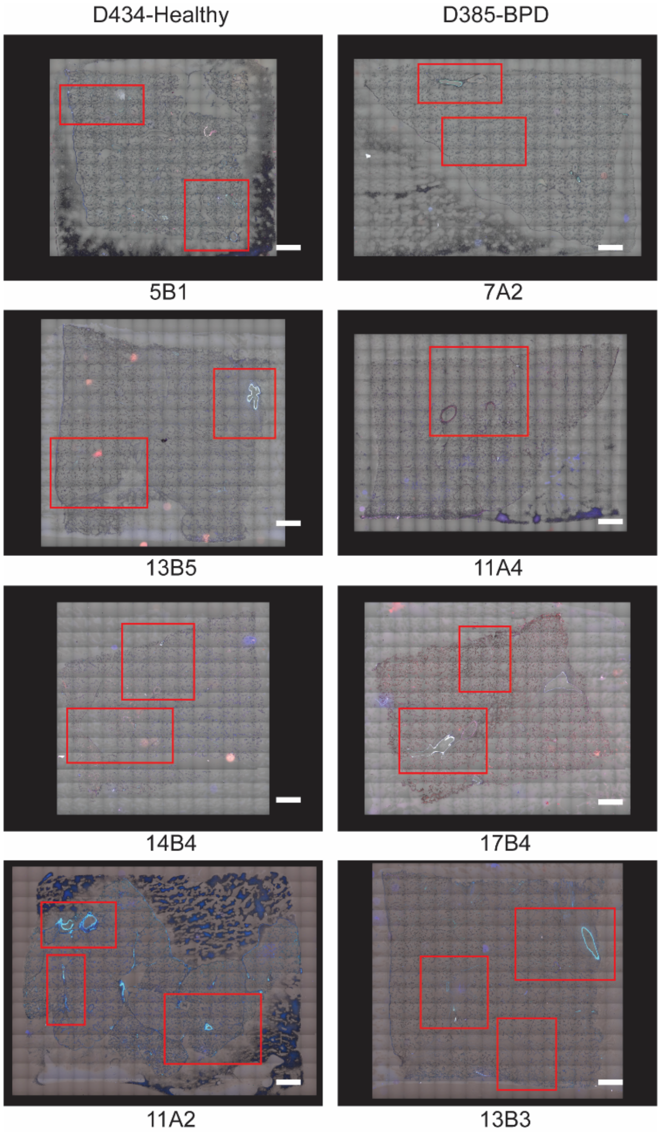

Figure S4. Autofluorescence images of each tissue analyzed section. Autofluorescence images showing the red, green, blue, and transmitted light. ROIs were selected for MALDI-MSI analysis at 35 µm containing bronchi, bronchioles, vessels, and alveolar parenchyma (red boxes).

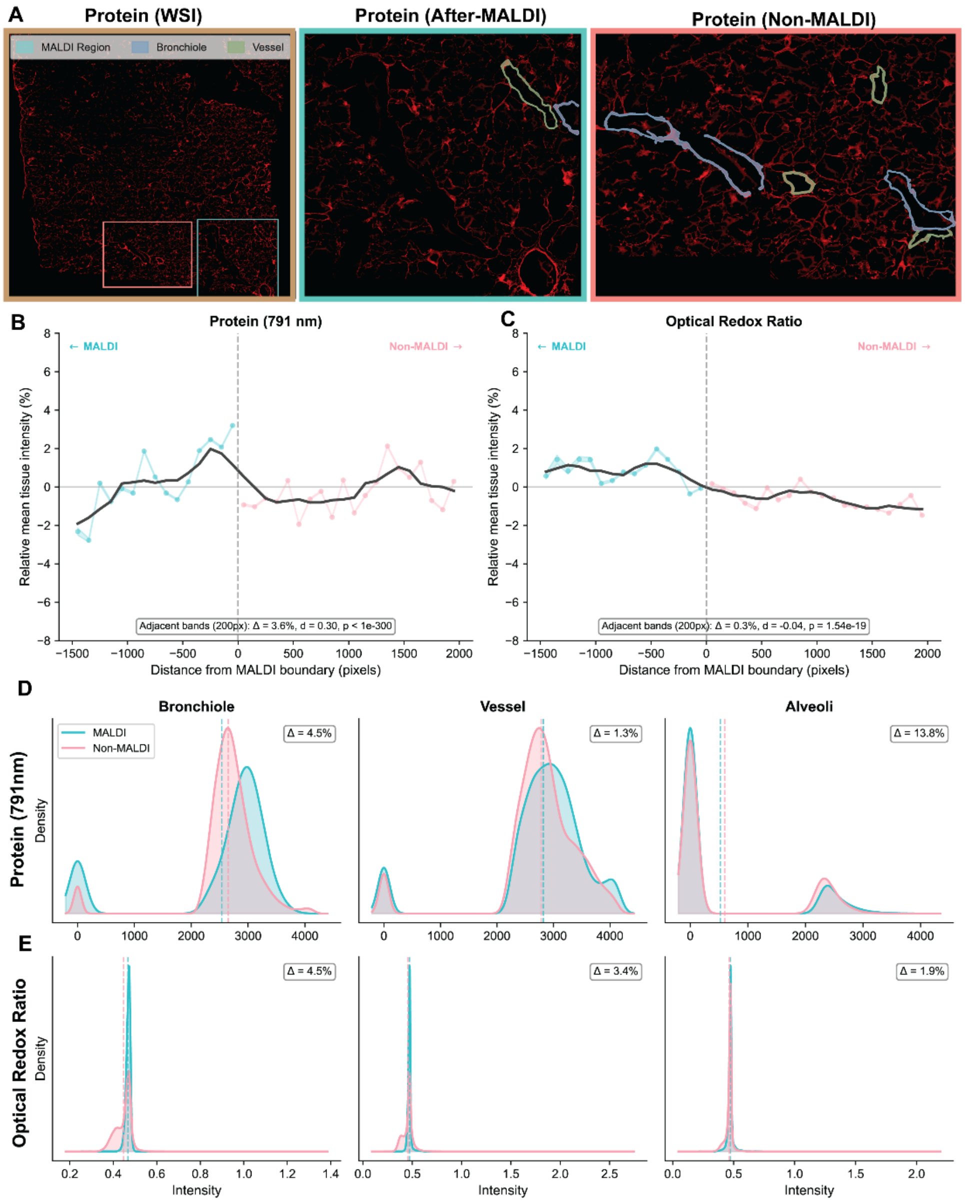

Figure S5. Prior MALDI-MSI processing does not alter subsequent U-FLIP fluorescence signals. **(A)** Full field-of-view SRS protein image (791 nm) of a single lung tissue section in which only the bottom-right quadrant (cyan outline) underwent MALDI-MSI acquisition prior to U-FLIP imaging. Bronchiole (steel blue) and vessel (sage green) annotations delineate matched functional tissue units (FTUs) within both the MALDI-processed and adjacent non-MALDI analyzed regions. **(B–C)** Boundary transition analysis for the SRS protein channel (B) and the optical redox ratio (C). Mean tissue intensity is plotted as a function of signed Euclidean distance from the MALDI boundary (dashed line; negative = inside MALDI region, positive = outside), binned in 100-pixel-wide bands spanning ±2,000 pixels. Colored points indicate raw band means; the solid line shows a five-band moving average; shading denotes 95% confidence intervals. A Welch's t-test of the 200-pixel bands immediately flanking the boundary yielded a 3.6% difference (Cohen's d = 0.30) for the SRS protein channel and a 0.3% difference (Cohen's d = −0.04) for the optical redox ratio. **(D–E)** Kernel density estimates of pixel-level intensity distributions for bronchiole, vessel, and alveolar parenchyma, comparing MALDI-processed (cyan) and non-MALDI (light red) regions for the SRS protein channel (D) and the optical redox ratio (E). Tissue-dense structures (bronchiole and vessel) show differences of 1.3–4.5%, and the alveolar optical redox ratio differs by only 1.9%, confirming that MALDI processing does not introduce a detectable artifact into U-FLIP measurements.

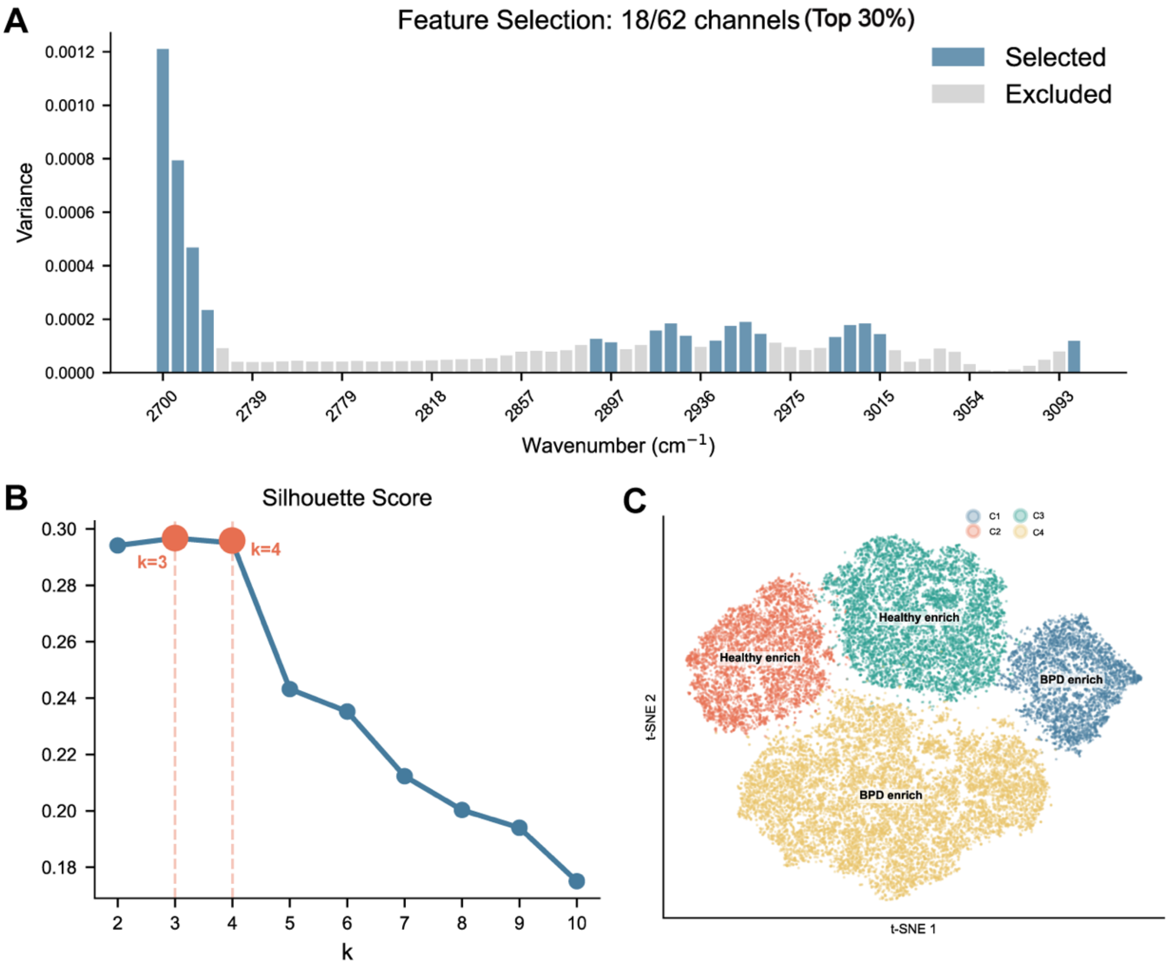

Figure S6. Feature selection and clustering optimization for hyperspectral SRS analysis of alveoli. (A) Variance-based feature selection retaining the top 30% of spectral channels (18 of 62) from the hSRS CH-stretching region. **(B)** Silhouette score across k = 2–10; k = 3 and k = 4 yield comparable scores, with k = 4 selected to better resolve spectrally distinct subpopulations with interpretable chemical signatures**. (C)** t-SNE embedding of the selected spectral features colored by k-means cluster assignment (k = 4), showing separation of healthy-enriched (C2, C3) and BPD-enriched (C1, C4) populations.

Tabel S1. All human lung tissue block IDs. Bronchopulmonary Dysplasia is abbreviated as BPD.

Donor ID	Block ID	Condition	Cause of Death	GA at Birth (wks)	Postnatal Age at Demise (mo)	Age Corrected for Prematurity (mo)	Histopathology
**D385**	LUL-13B3 LUL-17B4 LUL-13B5 LUL-7A2	BPD	BPD	25	7.9	4.4	Chronic lung disease of prematurity, reduced alveolarization, enlarged simplified airspaces, patchy early interstitial fibrosis and extension of smooth muscle into lobules, Focal bronchial squamous metaplasia
**D434**	LUL-11A2 LUL-11A4 LUL-14B4 LUL-5B1	No known lung disease	Cardiovascular/ natural causes	40	2.7	2.7	Normal lung structure and alveolar growth. Patchy minimal acute and chronic airway inflammation

Table S2. Student's T-test results comparing pixel intensities in each BPD functional unit to the corresponding healthy functional units. Performed within ROmics Processor.

Table S3. Student's T-test enrichment results comparing pixel intensities in each healthy functional unit to all other tissue regions. Performed within ROmics Processor.

Table S4. Student's T-test results comparing mean intensities of each BPD tissue functional unit (n=4) to the cooresponding healthy functional units (n=4). Performed within ROmics Processor.

Table S5. Student's T-test enrichment results comparing mean intensities of each functional unit (n=4) to all other regions. Performed within ROmics Processor.
